# Subretinal electrical stimulation preserves inner retinal function in RCS rat retina

**Published:** 2013-05-06

**Authors:** Vincent T. Ciavatta, Julie A. Mocko, Moon K. Kim, Machelle T. Pardue

**Affiliations:** 1 Rehab R&D Center, Atlanta VA Medical Center, Decatur, Georgia; 2 Department of Ophthalmology, Emory University, Atlanta, Georgia

## Abstract

**Purpose:**

Previously, studies showed that subretinal electrical stimulation (SES) from a microphotodiode array (MPA) preserves electroretinography (ERG) b-wave amplitude and regional retinal structure in the Royal College of Surgeons (RCS) rat and simultaneously upregulates *Fgf2* expression. This preservation appears to be associated with the increased current produced when the MPA is exposed to ERG test flashes, as weekly ERG testing produces greater neuroprotection than biweekly or no testing. Using an infrared source to stimulate the MPA while avoiding potential confounding effects from exposing the RCS retina to high luminance white light, this study examined whether neuroprotective effects from SES increased with subretinal current in a dose-dependent manner.

**Methods:**

RCS rats (n=49) underwent subretinal implantation surgery at P21 with MPA devices in one randomly selected eye, and the other eye served as the control. Naïve RCS rats (n=25) were also studied. To increase SES current levels, implanted eyes were exposed to 15 min per session of flashing infrared light (IR) of defined intensity, frequency, and duty cycle. Rats were divided into four SES groups that received ERG testing only (MPA only), about 450 µA/cm^2^ once per week (Low 1X), about 450 µA/cm^2^ three times per week (Low 3X), and about 1350 µA/cm^2^ once per week (High 1X). One eye of the control animals was randomly chosen for IR exposure. All animals were followed for 4 weeks with weekly binocular ERGs. A subset of the eyes was used to measure retina *Fgf2* expression with real-time reverse-transcription PCR.

**Results:**

Eyes receiving SES showed significant preservation of b-wave amplitude, a- and b-wave implicit times, oscillatory potential amplitudes, and post-receptoral parameters (Vmax and log σ) compared to untreated eyes. All SES-treated eyes had similar preservation, regardless of increased SES from IR light exposure. SES-treated eyes tended to have greater retinal *Fgf2* expression than untreated eyes, but *Fgf2* expression did not increase with IR light.

**Conclusions:**

The larger post-receptoral responses (Vmax), greater post-receptoral sensitivity (logσ), and larger oscillatory potentials suggest SES-treated eyes maintained better inner retinal function than the opposite, untreated eyes. This suggests that in addition to preserving photoreceptors in RCS rats, SES may also promote more robust signal transmission through the retinal network compared to the control eyes. These studies suggest that the protective effects of SES on RCS retinal function cannot be improved with additional subretinal current induction from the MPA, or the charge injection provided by ERG Ganzfeld flashes was not adequately mimicked by the flashing IR light used in this study.

## Introduction

The retina comprises a coordinated network of specialized cells that convert photon energy into chemical signaling between retinal neurons, and ultimately action potentials in ganglion cells. Retinal degenerative diseases such as retinitis pigmentosa (RP) and age-related macular degeneration (AMD) cause photoreceptor death and remodeling of inner retinal circuitry [1]. Such photoreceptor death and disrupted signal transmission result in irreversible vision loss as proper synaptic inputs to and action potentials from ganglion cells are compromised. These changes in retinal function are reflected in slower, attenuated electroretinography (ERG) responses in patients and animals with retinal degeneration.

Neuroprotection is a strategy for delaying or preventing photoreceptor death and subsequent vision loss from RP and AMD. Electrical stimulation applied via electrodes in the subretinal space or on the cornea has provided morphologic and/or functional neuroprotection in inherited retina degeneration animal models (dystrophic Royal College of Surgeon [RCS] rats [2,3], P347L transgenic rabbits [4], and P23H-1 rats [5]) and light-induced rat retinal degeneration models [6,7]. Preserved retinal function from retinal electrical stimulation may involve several mechanisms. One potential mechanism is through inducing growth factors known to have protective effects on photoreceptor survival and retinal function [8]. Retinal electrical stimulation has been shown to induce such factors [2,3,5], and in some cases, this induction was shown to mediate protective effects as specific antagonists of growth factor signaling cancelled or reduced protection from electrical stimulation [9,10]. Induction of antiapoptotic molecules is also associated with retinal electrical stimulation and, therefore, may play a role in the protective mechanism [6].

Separate from preservation, improvement in retinal and visual function was suggested by two retinal electrical stimulation clinical trials using subjects with RP. One trial tested subretinal microphotodiode array (MPA) retinal prostheses in subjects with late-stage RP and showed sustained visual acuity improvement in some subjects, suggesting improved retinal function several millimeters from the implantation/stimulation site [11]. The other trial involving subjects with RP treated for 6 weeks with 30 min/week transcorneal electrical stimulation showed significant improvement in the visual field area and scotopic b-wave [12]. There are no data from these trials to demonstrate the mechanism, but potential causes include improved function of extant photoreceptors, improved post-receptoral transmission of visual information through the retinal network, and placebo effect. Improved post-receptoral transmission is supported by the observation that ex vivo transretinal electrical stimulation of explanted RCS retina protected inner retinal neurons from apoptosis [13].

While investigating how subretinal electrical stimulation (SES) in the RCS rat preserves retinal function, researchers observed that ERG b-wave preservation depends on weekly stimulation from the MPA in response to ERG Ganzfeld flashes [2,14,15], as eyes treated with biweekly MPA stimulation were indistinguishable from controls or inactive device–implanted eyes [16]. This suggested total SES dose and/or more frequent administration may be critical for protective effects. Similarly, albeit improved retinal and visual function in the subjects with RP, the clinical trial [12] showed significant improvements in the ERG b-wave amplitude and visual field when suprathreshold, and not when sub-threshold, electrical stimulation (as determined with phosphene generation) was used, which also implies a dose–response relationship for protective effects. The purposes of this study were to determine if increasing the SES dose beyond that achievable with weekly Ganzfeld flashes (from ERG recordings) 1) could enhance preservation of retinal function and 2) is associated with further induction of *Fgf2* as this growth factor was shown to protect against photoreceptor loss in genetic [17] and light-induced [18] retinal degeneration rat models, preserved retinal function in RCS rats [19], and was selectively upregulated by SES whereas *Bdnf*, *Cntf*, *Fgf-1*, *Gdnf*, and *Igf-1* were unchanged [15].

## Methods

### Animals and experimental design

Pigmented dystrophic RCS rats from a homozygous breeding colony were obtained from Dr. Matthew LaVail (University of California San Francisco) and maintained at the Atlanta VA Medical Center. These animals possess a null mutation in the *Mertk* gene, which is necessary for circadian phagocytosis of shed photoreceptor outer segment (OS) disks by the retinal pigment epithelium (RPE) [20,21]. Mutations in this gene disrupt RPE phagocytosis, which leads to accumulation of used outer segment material, subsequent disruption of the OS-RPE interface, and eventual photoreceptor cell death. In this rat model, retinal degeneration begins around postnatal day (P)12, proceeds in a graded and preferential manner such that the inferior portion of the retina degenerates more rapidly compared to the superior region and rod photoreceptors die in much greater proportion than the cone photoreceptors, and almost all of the photoreceptors have degenerated by approximately P95 [22].

Animals were housed in a vivarium on 12 h:12 h light-dark cycle with standard room fluorescent lighting ranging from 25 to 200 lux. All animal procedures were approved by the Institutional Animal Care and Use Committee of the Atlanta Veterans Administration and adhered to the standards of the Association for Research in Vision and Ophthalmology Statement for the Use of Animals in Ophthalmic and Vision Research.

Since the main objective of the study was to test the hypothesis that neuroprotective effects depend on the SES dose, all surgically-treated eyes in this study received active MPAs. Although we have observed some outer nuclear layer (ONL) preservation with inactive devices (i.e., the MPA platform that lacks photodiodes) [2], neither in that study nor in the subsequent studies [15,16] did inactive devices have an effect on ERG b-wave amplitude at 4 weeks after implantation, whereas weekly SES resulted in significantly higher ERG b-wave amplitude at 4 weeks after implantation. Similarly, another experimental group showed less frequent SES (i.e., biweekly ERG of MPA-implanted eyes) did not preserve ERG b-wave amplitude compared to weekly SES [14]. We therefore allocated all surgically-treated rats to receive some level of weekly SES administration, believing there was sufficient evidence that SES was critical and hypothesizing that the effects were dose-dependent. To increase the dose, SES amplitude and duration were modulated above that from Ganzfeld flashes. MPA devices were implanted in the subretinal space of the RCS rats as before [23], and the eyes were exposed to infrared (IR) light. Light of infrared wavelength was chosen because the MPA device is maximally sensitive to about 800 nm light [24], and IR avoids potential photoreceptor preservation and growth factor induction in the RCS retina from prolonged white light stress [25].

Seventy-four RCS rats were divided into two groups: implanted rats, which received an electrically active subretinal MPA in one eye at P21 (n=49), and control rats, which did not undergo any surgical procedures (n=25). The MPA-implanted and control groups were further subdivided into groups for IR exposure (Table 1): MPA only, Low 1X, Low 3X, and High 1X. All animals received weekly ERGs beginning 1 week after implantation (P28) to provide stimulation to the MPA-implanted eyes and to evaluate retinal function in all eyes. Two days after the last ERG, the animals were euthanized, and a subset of these animals (15, control; 36 implanted [ten MPA only, seven Low 1X; six Low 3X; and ten High 1X]) was used for growth factor expression analysis. Because our previous studies tested a panel of growth factors (*Bdnf*, *Cntf*, *Fgf1*, *Gdnf*, and *Igf-1*) and found that *Fgf2* was selectively upregulated with SES [15], we focused on the effect of SES dose on *Fgf2* expression.

**Table 1:** SES treatment groups.

Light source	Estimated peak current density at retina - MPA interface (µA/cm^2^)	Duration (per week)
ERG	760	5.6 ms
IR (Low 1X)	450	18 s
IR (Low 3X)	450	54 s
IR (High 1X)	1355	18 s

The SES treatment groups are tabulated with an estimate of subretinal current density (i.e., SES amplitude) and duration. Current density was estimated as described in the Methods section. For Ganzfeld, duration was estimated from 200 microseconds/flash ×28 flashes. For IR, duration was estimated from (nominally, 1 ms of current transient/light flash) ×(number of light flashes).

### Microphotodiode array devices and surgery

The MPA used in this work has been described previously [16,26]. Briefly, the MPA is an iridium oxide-coated silicon wafer (1 mm diameter, 25 µm thick). One side of the MPA is composed of approximately 1250 square pixels (25 µm^2^ each) that contain a central stimulating photodiode each connected to a separate electrode. The other side is a single, common return electrode. Rats underwent monocular (random choice of which eye) implantation of the MPA in the subretinal space as described previously [23]. MPA devices were surgically placed in the superior subretinal space by gently rotating the eye inferiorly and creating an incision through all ocular layers, as previously described [23].

### Retinal electrical stimulation

Because MPA surfaces and surroundings (in this case, the subretinal milieu) form a capacitive interface that polarizes with continuous illumination and opposes charge injection, intermittent flashes must be shined on the photodiodes to induce current transients [27]. Weekly, flashed light came from two sources: ERG Ganzfeld (described below) and a custom-built IR light–emitting diode (LED) array (λ_max_ 873 nm, 44 nm full width at half height), essentially as described previously [24]. Maximum current density produced by the MPA from Ganzfeld and IR flashes was not measured in vivo, but can be estimated by integrating the product of the light power density function and the MPA responsivity function [24] over the interval of wavelengths provided by the respective sources. For simplicity, the Ganzfeld flash was assumed to be monochromatic at 555 nm, implying responsivity_Ganzfeld_=0.26 A/W, and for IR, all power was assumed at 873 nm, implying responsivity_IR_=0.345 A/W. Light power density (mW/cm^2^) at the retina (Ganzfeld, 2.773; low IR, 1.32; high IR, 3.96) was estimated from the product of the light power density at the cornea (measured with photometry), transmission through ocular media (0.75), and a geometric factor (0.088) determined for the rat eye [28]. Maximum stimulation currents were thus estimated as Ganzfeld, 760 μA/cm^2^; low IR, 450 μA/cm^2^; high IR, 1355 μA/cm^2^ by multiplying the light power density by the MPA responsivity. The total subretinal current in response to low and high IR was expected to be several hundred times greater than that from the Ganzfeld flash due to the much longer aggregate duration of exposure to the flashing stimulus (see below).

### Infrared light

In awake rats, the pupils were dilated (1% tropicamide, 1% cyclopentolate), and the rats were placed in a clear acrylic rodent restrainer. The IR stimulator was positioned 5 cm from the eye and delivered 20 or 60 mW/cm^2^ peak illumination at 20 Hz with 10 ms/flash (see Table 1). The 20 Hz frequency was based on reports of neuroprotective effects from transcorneal electrical stimulation in rat retinal degeneration models [3,6]. Fifteen-minute stimulation treatments were done once per week in a dimly lit room.

### Ganzfeld flash

All rats in the study had weekly ERG recordings to measure retinal function. A consequence of ERG recording is increased SES as Ganzfeld flashes produce transient current from the subretinal MPA. The number of flashes and stimulus levels are provided below.

### Electroretinography recordings and analysis

Rats were dark-adapted overnight or for at least 4 h, and all preparations were done under dim red illumination. Rats were anesthetized with ketamine (80 mg/kg) and xylazine (7.5 mg/kg) and placed on a heating pad to maintain body temperature at 37 °C. Corneas were anesthetized with 0.5% tetracaine and pupils dilated with 1% cyclopentolate and 1% tropicamide. Responses were recorded with nylon fibers embedded with silver particles placed across both corneas and wetted with 1% methylcellulose. Platinum needle electrodes were placed in the cheeks for reference and in the tail to serve as ground. Under dark-adapted conditions, −3.4 to 1.4 log cd s/m^2^ flashes were presented by a Ganzfeld dome. The Ganzfeld was calibrated with a photometer (International Light, IL1700 radiometer, Newburyport, MA) using a scotopic filter and thus, luminance units are scotopic candelas. With increasing flash stimuli, three to ten responses were averaged, and the inter-flash interval was increased from 2 to 70 s. All responses were filtered (1–1500 Hz) and stored on a commercial ERG system (UTAS 3000; LKC Technologies, Gaithersburg, MD) for later analysis.

ERG records from all animals at 4 weeks post-op were analyzed because earlier studies [2,15] showed an ERG b-wave amplitude difference (i.e., preservation in SES-treated retina) beginning 4 weeks after surgery, whereas earlier time points did not show a difference between SES-treated eyes and controls. Briefly, the following parameters were derived from the ERG recordings, as previously described [29]. Scotopic a-wave and b-wave amplitudes and implicit times were measured from raw waveforms. The saturated rod-driven post-receptoral response (Vmax) and log of 1/2 flash intensity required to reach Vmax (logσ) were derived from fitting the Naka-Rushton equation to the dark-adapted b-wave intensity-response plot [30]. Finally, oscillatory potentials (OPs) 1 through 6 and their implicit times were derived from filtering original waveforms using a fifth-order Butterworth filter with bandpass 60–235 Hz. Amplitudes were measured from the trough immediately preceding the peak of each OP for OPs 1–6. Implicit time for each OP was measured from the flash onset to the peak of the OP.

### Molecular analysis

Two days after the final ERG, the animals were euthanized with an intraperitoneal injection of 100 mg/kg pentobarbital. Tissue preparation and growth factor expression analyses were performed as previously described (implanted n=33, control n=15) [15]. Briefly, the eyes were enucleated, the cornea and lens were removed, and a 2.3-mm diameter portion of the retina over the implant (or an equivalent location in non-implanted eyes) was dissected and used for RNA isolation. Total RNA was recovered (Qiagen RNeasy Micro kit with DNase Treatment, Qiagen, Valencia, CA) following the manufacturer’s instructions. One hundred nanograms of RNA was converted to cDNA (Qiagen QuantiTect Reverse Transcriptase). cDNA was diluted 20 fold, and 5 µL of diluted cDNA was used in real-time PCR reactions with 200 nM (*Fgf2*, *18S*) of each forward and reverse primers [15]. Samples were run in triplicate, and the average cycle threshold (Ct) was calculated. Using 18S as an internal standard, relative growth factor expression was calculated from the average PCR cycle thresholds (Ct) taking into account the efficiency of amplification as described in [31].

### Statistical analysis

**Figure 1:**
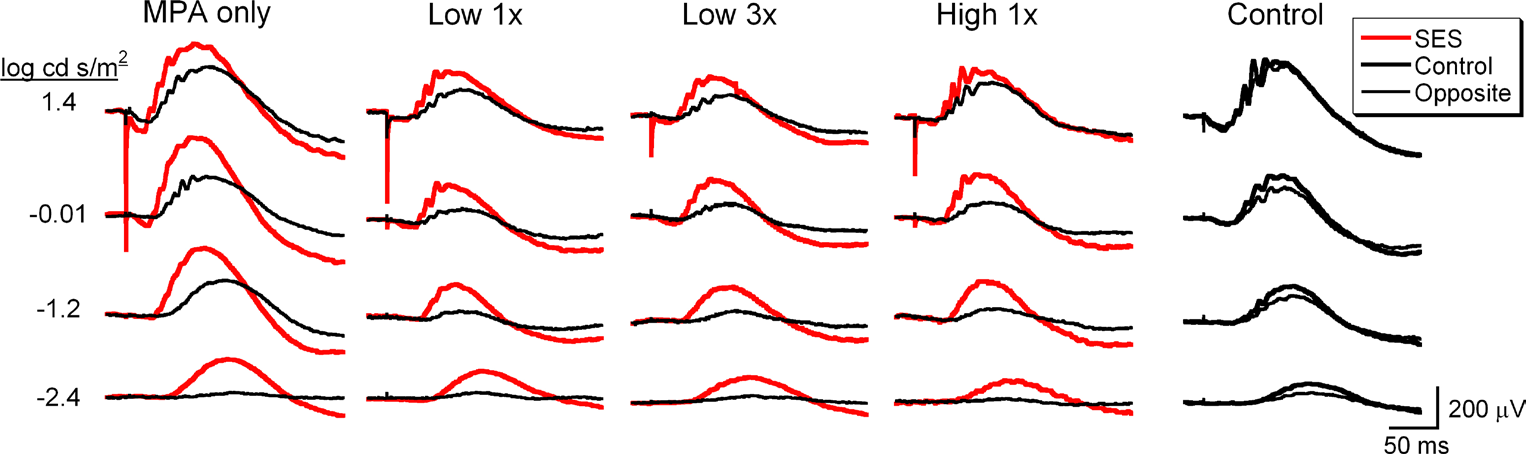
Representative electroretinogram waveforms across flash stimuli recorded from implanted and contralateral eyes from each subretinal electrical stimulation (SES)–treated group and a naïve rat at 4 weeks post-op. Waveforms in the subretinal electrical stimulation (SES)–treated eyes were greater than in the opposite eyes in each group. Waveforms in the right and left eyes of the naïve rats were nearly identical. The large, fast, negative peak recorded from microphotodiode array (MPA)-implanted eyes is an “implant spike” caused by a rapid charge injection from the MPA in response to the brighter flash luminances.

To minimize variability between litters of RCS rats from multiple breeding pairs (see Figure 1 representative waves), all data were examined as relative values (i.e., a ratio of the treated eye to the opposite, unimplanted eye or for naïve, control rats, the left eye was considered the “treated” eye). For responses analyzed across flash stimuli (a-wave, b-wave, and OP amplitudes and implicit times), values were normalized to the maximum flash value of the opposite eye, and a two-way repeated-measures analysis of variance (ANOVA) was used to compare responses across flash stimuli and treatment groups (SigmaStat 3.5, Systat Software, Inc., San Jose, CA). ERG parameters (max a-wave, max b-wave, Vmax, logσ, and OPs 1–6 max) were analyzed using one-way ANOVA (SigmaStat 3.5). Since no differences in ERG parameters were found between the various SES treatment groups, Student *t* tests or the Mann–Whitney rank-sum test, if normality failed, was performed to compare all MPA-implanted eyes versus controls (SigmaStat 3.5). Similarly, because no differences were found among all unoperated eyes, regardless of IR treatment, “control” data were averaged from all unoperated eyes. One-way ANOVAs with post hoc Holm-Sidak multiple comparison tests were used for *Fgf2* expression analyses. Interaction effects of ANOVAs are reported unless otherwise indicated. Error bars in all graphs represent ±standard error of the mean (SEM).

## Results

### Retinal function

**Figure 2:**
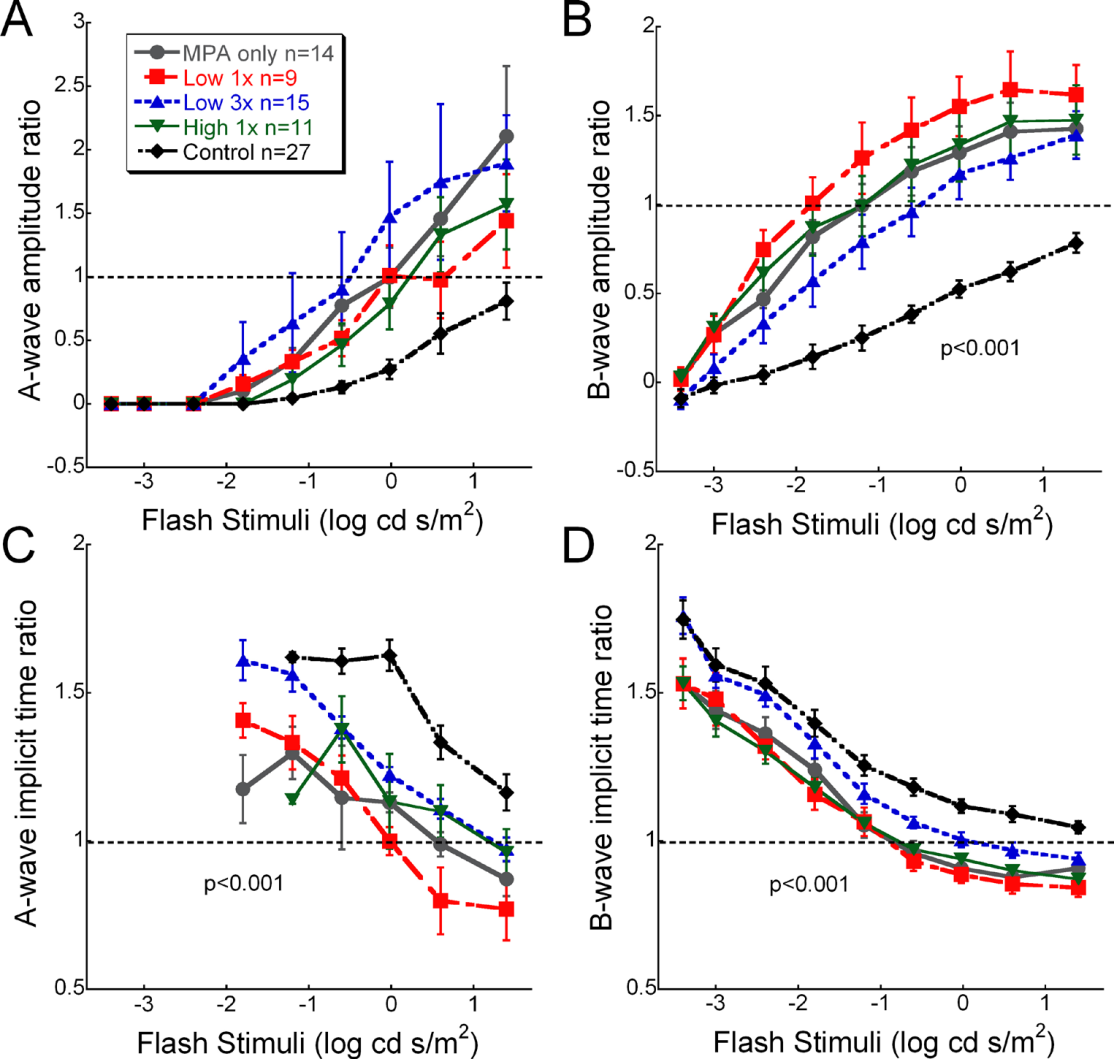
Average relative amplitude and implicit time for the a- and b-wave across flash stimuli for each treatment group. Each value is a ratio of the treated eye/maximum flash value of the control eye. **A**: A-wave amplitude ratios tended to be higher in eyes with subretinal electrical stimulation (SES) treatment, but this did not reach statistical significance. **B**: The b-wave amplitude ratios were significantly greater in eyes receiving SES compared to the naïve controls (repeated ANOVA F(36, 699)=6.018, p<0.001). **C**: The a-wave implicit time ratios were significantly faster than the control group (repeated ANOVA main effect F(4,290)=9.878, p<0.001). **D**: The b-wave implicit time ratios were also significantly faster than the control group (repeated ANOVA main effect F(4,699)=7.865, p<0.001). Error bars represent ±SEM.

Overall, eyes receiving SES via MPA showed better-preserved ERG waveforms compared to non-implanted eyes (Figure 1). All eyes receiving SES had greater amplitude a- and b-waves compared to controls. Figure 2A shows a strong trend for a larger a-wave amplitude ratio across all flash stimuli. However, variability in the small a-wave response at this stage of degeneration prevented this from becoming statistically significant. As shown in Figure 2B, the b-wave amplitude ratio was significantly greater in all eyes receiving SES treatment compared to the naïve controls (repeated ANOVA, F(36, 699)=6.018, p<0.001). The mixed rod and cone b-wave response to bright flashes was about 50% greater than the response from the control eyes. Furthermore, eyes receiving SES had faster a- and b-wave implicit time ratios compared to controls (Figure 2C,D; repeated ANOVA main effect, F(4,290)=9.88, p<0.001 and F(4,699)=7.87, p<0.001).

**Figure 3:**
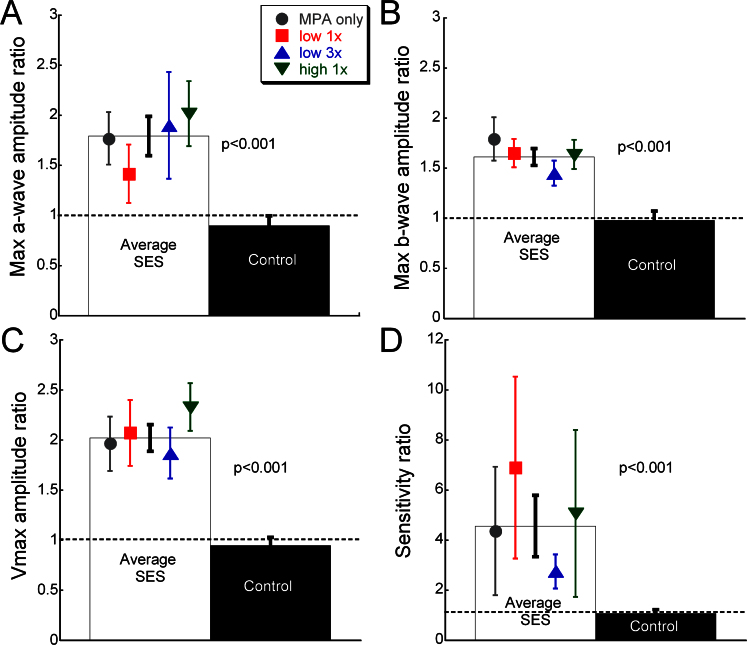
Assessment of electroretinogram parameters for each of the treatment groups at 4 weeks post-implantation. Symbols represent the different microphotodiode array (MPA) and infrared (IR)-treated groups while the white bar represents the average of all the subretinal electrical stimulation (SES)-treated eyes, which were not significantly different. **A**: Amplitude ratios for maximum a-wave (**A**), maximum b-wave (**B**) and Vmax (**C**) were significantly greater in SES-treated eyes compared to the controls (p<0.001). **D**: Logσ showed that retinal sensitivity was greatest in eyes treated with SES compared to the controls (p<0.001). Animal numbers are the same as listed for Figure 2. Error bars represent ±SEM.

After the ERG parameters max a- and b-wave waves, Vmax and logσ, were analyzed, the MPA-implanted eyes with or without IR treatment were not significantly different (shown by symbols in Figure 3). Thus, these groups were combined and statistically compared to the control group. This analysis showed that maximum a- and b-wave amplitude ratios were significantly greater by about 50% to 100% in all eyes receiving SES (p<0.001; Figure 3A,B). Furthermore, scotopic post-receptoral function and retinal sensitivity as measured with Vmax and logσ, respectively, were also significantly greater with SES treatments (p<0.001; Figure 3C,D). Vmax in the SES-treated eyes was consistently twice as large as in the control eyes. Logσ showed more variability, but also greater preservation with three- to sixfold greater sensitivity after SES treatment for 4 weeks.

**Figure 4:**
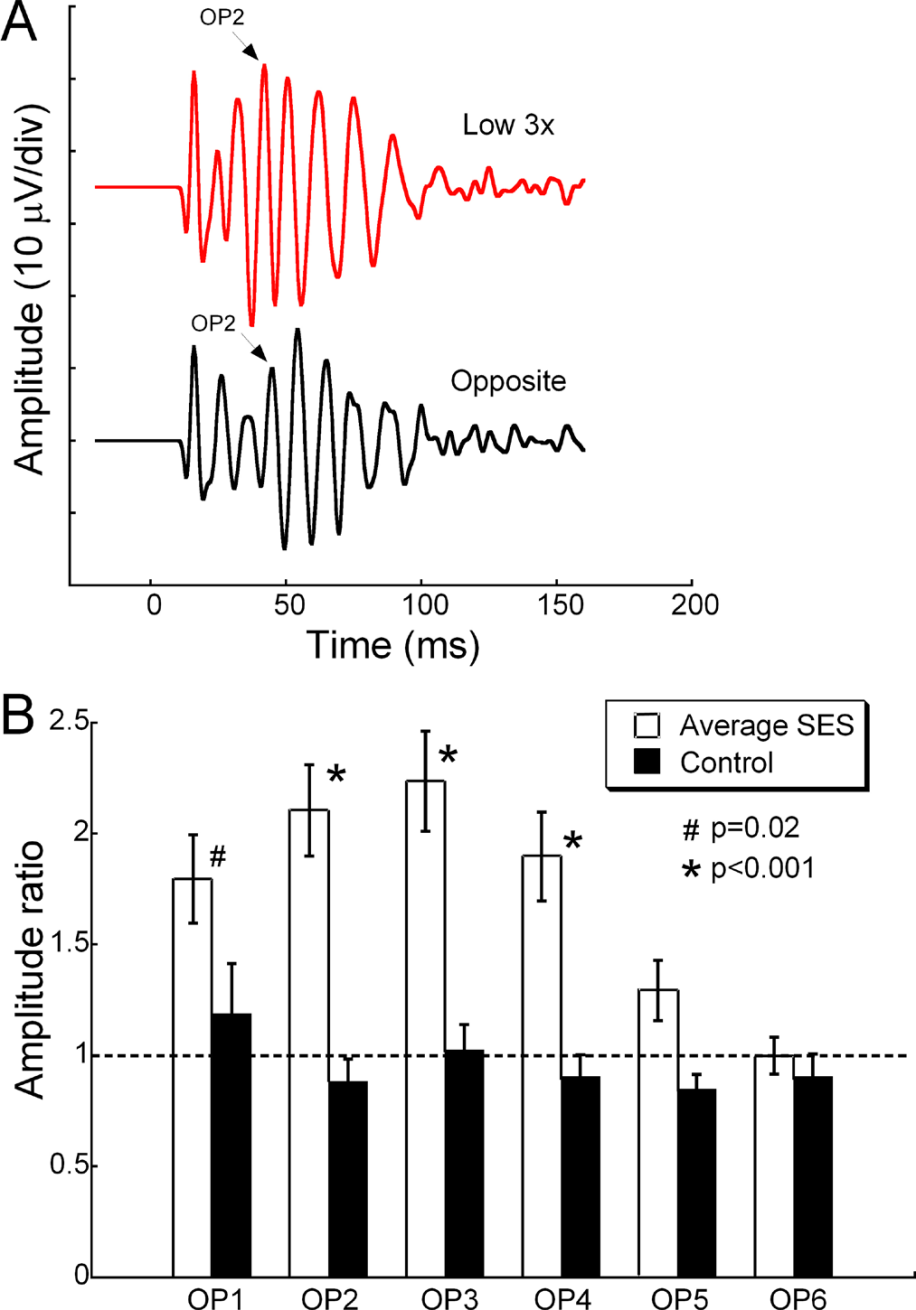
Subretinal electrical stimulation (SES) preserved inner retinal function as measured by oscillatory potential (OP) amplitudes. **A**: Representative oscillatory potential (OP) waveforms in response to a bright flash (1.4 log cd s/m^2^) at 4 weeks, showing larger amplitude responses in the subretinal electrical stimulation (SES)-treated eye. **B**: Maximum OPs 1–6 amplitude ratios after 4 weeks of SES. Treatment with SES resulted in larger OPs 1–4 amplitude ratios in SES-treated eyes compared to the controls (p=0.016 for OP 1 and p<0.001 for OPs 2–4). The later OP amplitude ratios (OP 5 and OP 6) were not different between SES-treated and control eyes. Error bars represent ±SEM.

Examination of inner retinal function via the OPs showed increased amplitudes with SES treatment across the different OPs. Figure 4A shows representative OP waveforms from an IR-stimulated eye (Low 3x) with larger OP amplitudes versus the opposite eye in response to a bright flash (1.4 log cd s/m^2^). There was also a trend for increased OP implicit time in SES-treated eyes versus controls, but this was not significant (data not shown). Figure 4B shows the maximum OP amplitude ratios at 4 weeks for OPs 1–6. The SES-treated eyes had significantly larger OP 1 amplitude ratios (p=0.016) and OPs 2–4 amplitude ratios (p<0.001). Note that the preservation effect was not observed in OP 5 and OP 6.

### *Fgf2* expression

**Figure 5:**
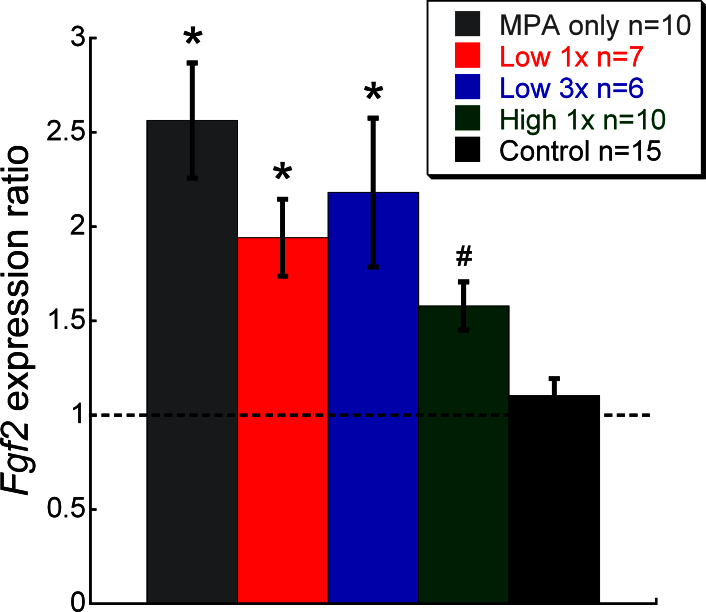
Relative *Fgf2* mRNA in retina among the different treatment groups after 4 weeks of subretinal electrical stimulation. Subretinal electrical stimulation (SES)–treated eyes in the microphotodiode array (MPA) only, Low 1X, and Low 3X *g*roups had greater *Fgf2* expression than the control group (* symbol). *Fgf2* expression in the MPA-only group was also greater than expression in the High 1X *g*roup (# symbol, p<0.05 post-hoc Holm-Sidak test for all statistical tests). Error bars represent ±SEM.

Real-time reverse-transcription PCR analysis showed significant differences in *Fgf2* expression among the treatment groups (ANOVA, F=8.69 (*F_crit_*=2.6), p<0.001). Significant *Fgf2* induction occurred in the SES-treated eye in three of the four SES-treated groups. However, *Fgf2* induction in retinas receiving the highest amplitude SES was not significantly different from the retinas in the control rats, but was significantly lower than induction in the MPA-only group (Figure 5; p<0.05, Holm-Sidak post-hoc analysis).

## Discussion

This study was undertaken to test the hypothesis that protection of retinal function in RCS rats by SES is dose dependent. Because the objective of the study was to test the hypothesis that neuroprotective effects depend on SES dose, and previous studies performed 4 weeks after surgery showed no differences in ERG parameters between eyes implanted with inactive devices (i.e., an MPA platform that lacks photodiodes) and non-surgical eyes [15,16], whereas weekly SES consistently resulted in significantly higher ERG b-wave amplitude, all surgically-treated eyes in this study received active MPAs. Consequently, to control for the effects of IR light flashes in the absence of SES, eyes without implants were used. Results indicate neither protection of retinal function nor expression of *Fgf2* depends on the SES dose. Although preservation was not increased with increasing SES, the results confirm that SES provides preservation of retinal function and is associated with increased *Fgf2* expression. Additionally, these experiments demonstrate that SES may preserve and enhance inner retinal function.

### Possible reasons for lack of a dose response

Results showed that increasing the SES dose did not result in additional preservation of ERG parameters or *Fgf2* expression. A potential dose response effect was inferred from previous studies where eyes receiving weekly SES clearly showed greater b-waves than eyes implanted with non-active devices [2,15] and eyes implanted with MPAs that received biweekly Ganzfeld flashes [14]. That no dose–response effect was observed in the present study may be due to several factors.

First, the maximal neuroprotective effect from SES may have been reached, and no further protection is possible in the RCS rat. This study was designed to try to optimize and improve SES effects and thus tried only to increase current levels. Use of lower SES may have produced a dose response that peaked at the level seen here and reported previously [2,14,15].

Second, the IR illumination used to increase current magnitude and duration may have created a stimulus from the MPA that was too dissimilar from that produced by ERG Ganzfeld flashes to be useful. Specifically, the ERG bright (xenon bulb) flashes have a duration between about 50 and 200 μs, whereas the IR flashes used in this study were 10 ms. Duration of charge separation within the MPA, and thus current induced in the surrounding environment, is limited to about 0.1 to 1 ms, whereas longer duration flashes do not produce sustained currents in the surrounding medium but instead form a capacitive interface between the MPA and surrounding environment that slowly diminishes as MPA internal charge separation is shunted within the MPA device [27]. The estimates of stimulus duration in Table 1 reflect this; 1 ms is used as the duration of stimulus current per flash (instead of the 10 ms flash duration). Consequently, retinas treated with SES from Ganzfeld flashes are expected to experience a burst of subretinal current during flash duration (about 50 to 200 μs) and upon flash offset, a similar burst of the subretinal current of opposite polarity, approximating a charge balanced, biphasic, sine wave. However, subretinal current from the IR stimulation provided in this study was expected to burst as with Ganzfeld flashes, but cease at approximately 1 ms, reverse polarity as the charge was shunted within the MPA device, and then increase rapidly when the light flash ended approximately 9 ms later. Further, the stimulus from Ganzfeld flashes occurred as single pulses separated by tens of seconds (dark-adapted) or at 2 Hz for 12.5 s (light-adapted), whereas stimulus from the IR flashes occurred at 20 Hz.

The importance of stimulation parameters for neuroprotection in the eye has been demonstrated previously. For example, transocular stimulation at 50 Hz had a greater ONL-preserving effect than 20 Hz on light-induced photoreceptor degeneration [6], and retinal ganglion cell preservation after axotomy depended on stimulus duration [32]. Thus, if the waveform, duration, or interval provided by IR could not trigger biologic effects equivalent to ERG flashes (e.g., channel opening, cellular stress response), then the effect of increased intensity or increased total stimulus duration could have been compromised. Future studies examining SES effects may need to match parameters known to preserve retinal function.

Third, since Ganzfeld flashes trigger phototransduction and consequent retinal network activity along with SES in MPA-implanted eyes, whereas IR flashes trigger only SES, simultaneous phototransduction may be a necessary component of the SES protective mechanism.

Fourth, rats exposed to IR were awake and restrained, whereas those receiving only ERG were anesthetized. Although the rats were acclimatized to the restraint, this could have induced a stress response that exacerbated retinal function loss. Additionally, IR stimulation of the implant may not have been optimal in the awake rats, which were able to turn their heads and eyes.

Fifth, possible heating of retinal tissues from IR absorption cannot be overlooked. Retinal heating was not measured in our study. Because ERG amplitudes and histology were not disturbed in the IR-treated retinas, it is unlikely potential retinal heating caused any tissue damage. However, extrapolating from Sailer et al.’s work [33] in which the in vivo temperature of the rabbit retina was measured as a function of continuous exposure to 0.421 to 168 mW/cm^2^ of 830 nm light for 2 min, the 20 and 60 mW/cm^2^ light used in our study might be expected to cause up to a 1 °C and 3.5 °C increase, respectively, if the IR was applied continuously. However, flashing light, as used here, is expected to reduce the heating effect [34], whereas the smaller rat eye is expected to transmit more light power to the retina and thus increase the heating effect. Further, we compared the IR used in this study to the near IR light used on pigmented rat eyes in study by Eells et al. [35], in which no thermal effects, but protection against methanol-induced retinal degeneration were observed. Specifically, the maximum permissible heating based on American National Standards Institute (ANSI) standards for human eye exposure [34] were calculated for both studies. These calculations suggest the heating in study by Eells et al. and our 20 mW/cm^2^ treatment are expected to exceed the maximum permissible heating standard equivalently. Thus, similar to extrapolation from the Sailer et al. studies [33] of IR heating in the rabbit retina, comparison to the Eells et al. study suggests our 20 mW/cm^2^ treatment produced negligible heating, whereas the 60 mW/cm^2^ treatment would be expected to produce more heat. We therefore infer that the IR light used in this study could have caused some non-damaging retinal warming, and that such warming could have altered the electrical stimulation effects.

### Significance of preserved electroretinography parameters

Results from this study showed significant preservation of post-receptoral ERG parameters in all SES-treated eyes, irrespective of SES dose. Higher Vmax in SES-treated retina implies greater maximal transmission of signals from rod photoreceptors to bipolar cells. Together, increased b-wave and Vmax amplitude could reflect greater signal input from photoreceptors (increased a-wave), better propagation of the signal across the photoreceptor-to-bipolar cell synapse, and better bipolar cell function. Because a-wave amplitude tended to be higher in SES-treated eyes, but was not significantly different from opposite eyes, increased Vmax likely reflects improved post-receptoral parameters, although contribution from improved rod function (a-wave) should not be totally ignored. That logσ was lower in the SES-treated eyes coupled with higher Vmax again indicates more efficient signal propagation (more bipolar cell activity per photon). Finally, higher OP amplitudes reflect receptoral and post-receptoral functions. The first, OP 1, largely reflects receptoral function [36] and thus is consistent with the trend for higher a-wave amplitudes noted for all SES-treated eyes. However, OPs 2–4 largely reflect the activity of action potential–dependent third-order inner retinal neurons [37]. Greater amplitude of these middle OPs in SES-treated eyes therefore suggests preserved inner retinal function in the degenerating retina is a consequence of SES.

### *Fgf2* expression

Piercing trauma of the rat eye, similar to that during MPA implantation surgery, is known to increase *Fgf2* expression [17]. Although such induction or administration of FGF2 can rescue photoreceptors in the RCS rat retina [16,38,39], evidence that FGF2 restores the disrupted outer segment phagocytosis in RCS rat RPE is limited to RPE culture experiments [40]. Thus, the FGF2 protective effects of the RCS retina likely reflect a generalized photoreceptor rescue phenomenon rather than rescue of deficient RPE phagocytic function [20,21].

Beyond acute trauma-induced *Fgf2* upregulation, SES was shown to increase and prolong *Fgf2* upregulation as MPA-implanted RCS eyes had higher *Fgf2* expression than inactive device–implanted eyes out to 4 weeks post implantation. Moreover, growth factor induction has been associated with protective effects of electrical stimulation on retinal structure and function [6,15,32]. In one case of transcorneal electrical stimulation, protective effects were shown to depend on insulin-like growth factor 1 (IGF-1) induction [9] as protective effects were eliminated by an IGF-1 receptor antagonist, and our studies to antagonize FGF2 signaling suggest FGF2 is partly responsible for the protective effects of SES on retinal (ERG b-wave amplitude) and visual (optokinetic tracking) function in the RCS rat [10]. However, in the Mer knockout mouse model, electrical stimulation increased growth factor expression, but retinal functional or structural preservation was not observed [29], suggesting that this model is not responsive to FGF2 or the murine homolog for the *Mertk* mutation may not be as responsive to neuroprotective strategies [41]. In the present study, *Fgf2* mRNA did not increase with SES dose, but was increased in retinas receiving SES, except the highest dose group (High 1X). That the highest dose group did not show a significant increase in retinal *Fgf2* message compared to the control retina but showed similar post-receptoral parameters as the other dosed groups suggests the following: 1) FGF2 is not the sole factor promoting neuropreservation from SES, and other as-yet-unidentified mechanisms (e.g., local leukemia inhibitory factor activation triggered by ligand release from SES) are central to the protective effects, and 2) lower amplitude SES, such as that produced during lower luminance Ganzfeld flashes, is needed for *Fgf2* induction while higher amplitude SES can be counter-productive for *Fgf2* induction.

The *Fgf2* induction in the High 1X *g*roup was not significantly different from the controls and significantly less than in the MPA-only group. This is most likely due to retinal heating and/or higher SES current density as these are the most notable differences between the High 1X and other treatment groups. Our estimates of retinal heating (see above), based on the published thermal effects of IR [33,35], suggest a possible slight temperature increase in all IR-treated eyes, with the largest temperature increase expected in the High 1X *g*roup. Whether this warming could affect *Fgf2* expression is not known. Interestingly, heat treatment of RPE has been shown to decrease angiogenic activity [42], and since FGF2 is a potent angiogenic factor, decreased *Fgf2* expression from retinal warming seems plausible. Regarding possible effects of higher SES current density, if the higher current damaged cells in the electric field, attenuated *Fgf2* might be expected if the damaged cells were the source of *Fgf2*. However, the estimated current density of about 1355 µA/cm^2^ in the High 1X *g*roup is about 1/100^th^ of the retinal damage threshold current density determined by Butterwick et al. [43] for a similar sized electrode and pulse duration, and thus, it seems unlikely the higher current density alone could have damaged cells responsible for the elevated *Fgf2*. Perhaps the combined effects of retinal warming and the higher current together attenuate *Fgf2* expression.

### Summary

This study examined a possible dose–response relationship between SES and retinal function preservation. Results showed that SES of the RCS rat retina can affect and preserve post-receptoral retinal function, but that preservation was not dependent on the SES dose provided in this study. The lack of a dose–response effect could be due to several factors, including 1) a saturating SES dose is achieved from Ganzfeld light flashes and no further functional preservation from SES is possible, or 2) preservation may depend on SES dose, but the effect was not adequately tested because the IR stimulus did not provide an adequate mimic of SES from Ganzfeld flashes, and/or other confounds such as retinal warming due to IR absorption masked additional neuroprotective effects. Doubling the current above that used previously [2,14,15] attenuated *Fgf2* mRNA induction but did not diminish preservation of post-receptoral function, suggesting other factors, in addition to FGF2 signaling, may promote neuroprotection from SES. Learning how to control these neuroprotective effects is important for subretinal prosthetic development so retinal health and signal transmission are optimally controlled. Furthermore, the positive effects on inner retinal function are consistent with implanting subretinal electronic retinal prosthetics early in the course of degeneration in candidate patients to precede and perhaps reduce extensive retinal remodeling.
